# The Interplay between Gut Microbiota and Diet-Induced Kidney Protection

**DOI:** 10.34067/KID.0000001219

**Published:** 2026-05-26

**Authors:** Francisco Lopes, Daniel Martinez-Martinez, Martin R. Späth, K. Johanna R. Hoyer-Allo, Sebastian Strubl, Sadrija Cukoski, Luisa Knieps, Susanne Brodesser, Heike Göbel, Günter Schwarz, Bernard M. van den Berg, Ton J. Rabelink, Bernhard Schermer, Thomas Benzing, Roman-Ulrich Müller, Andreas Beyer, Filipe Cabreiro, Felix C. Koehler

**Affiliations:** 1 Cologne Excellence Cluster on Cellular Stress Responses in Aging-Associated Diseases (CECAD), Faculty of Medicine and University Hospital Cologne, University of Cologne, Cologne, Germany; 2 Institute of Clinical Sciences, Imperial College London, Hammersmith Hospital Campus, London, United Kingdom; 3 Department II of Internal Medicine and Center for Molecular Medicine Cologne, Faculty of Medicine and University Hospital Cologne, University of Cologne, Cologne, Germany; 4 Institute for Pathology, Diagnostic and Experimental Nephropathology Unit, Faculty of Medicine and University Hospital Cologne, Cologne, Germany; 5 Institute of Biochemistry, Faculty of Mathematics and Natural Sciences, University of Cologne, Cologne, Germany; 6 Department of Internal Medicine (Nephrology) and Einthoven Laboratory of Vascular and Regenerative Medicine, Leiden University Medical Center, Leiden, The Netherlands; 7 Center for Rare Diseases Cologne, Faculty of Medicine and University Hospital Cologne, University of Cologne, Cologne, Germany; 8 Department of Nephrology, Medical Faculty, University Hospital Düsseldorf, Heinrich-Heine-University Düsseldorf, Düsseldorf, Germany

**Keywords:** AKI, acute renal failure, cell survival, ischemia, ischemia–reperfusion, ischemic renal failure, metabolism, proximal tubule

## Abstract

**Key Points:**

- Beneficial diets changed the composition of gut microbiota in an ischemia–reperfusion injury‑dependent manner in rodents.
- Taxonomic and functional profiling revealed a central role of *Lachnospiraceae*, a main producer of microbially derived short-chain fatty acids.
- Comparative bulk transcriptomics suggested the metabolic use of these bacterial products as an additional energy source in kidneys of protected mice.

**Background:**

On one hand, dietary interventions are known for their pivotal role in regulating diversity, composition, and function of the gut microbiome. On the other hand, specific diets show an immense potential in preventing kidney injury from various damaging stimuli in rodents, and recent findings, in turn, highlight a central role of gut microbiota in kidney health and disease.

**Methods:**

Three protective dietary regimens—a fasting-mimicking diet, a diet depleted in sulfur-containing amino acids, and caloric restriction—were examined in parallel in a rodent model of ischemia–reperfusion injury (IRI). To delineate the diet-induced effect on gut microbiota in response to ischemic kidney damage, we used comparative shotgun metagenomics for taxonomic and functional profiling. We further examined the renal metabolic response using comparative transcriptomics to unravel the interplay between gut microbiota and kidney protection.

**Results:**

Beneficial dietary preconditioning strategies changed the composition of gut microbiota in an IRI-dependent manner. Using ternary plots to investigate the role of dietary interventions over time before and after ischemic insult, we detected a central role of *Lachnospiraceae* that commonly expanded in response to renal IRI in dietary preconditioned mice. Further functional profiling of gut microbiota in our model revealed an increase in plasma levels of bacterial-derived short-chain fatty acids in diet-induced kidney protection. Comparative bulk transcriptomics in our model, in turn, pointed toward the metabolic use of these bacterial-derived short-chain fatty acids in kidneys of protected mice.

**Conclusions:**

Because proximal tubules lack sufficient glycolytic capacity, products of microbial metabolism may serve as an additional energy source to fulfill their high demands when withstanding ischemic damage. Our data shed light on a close interplay between gut microbiota and diet-induced kidney protection calling for further research at the crossroads of microbiology, metabolism, and molecular nephrology.

## Visual Abstract

**Figure:**
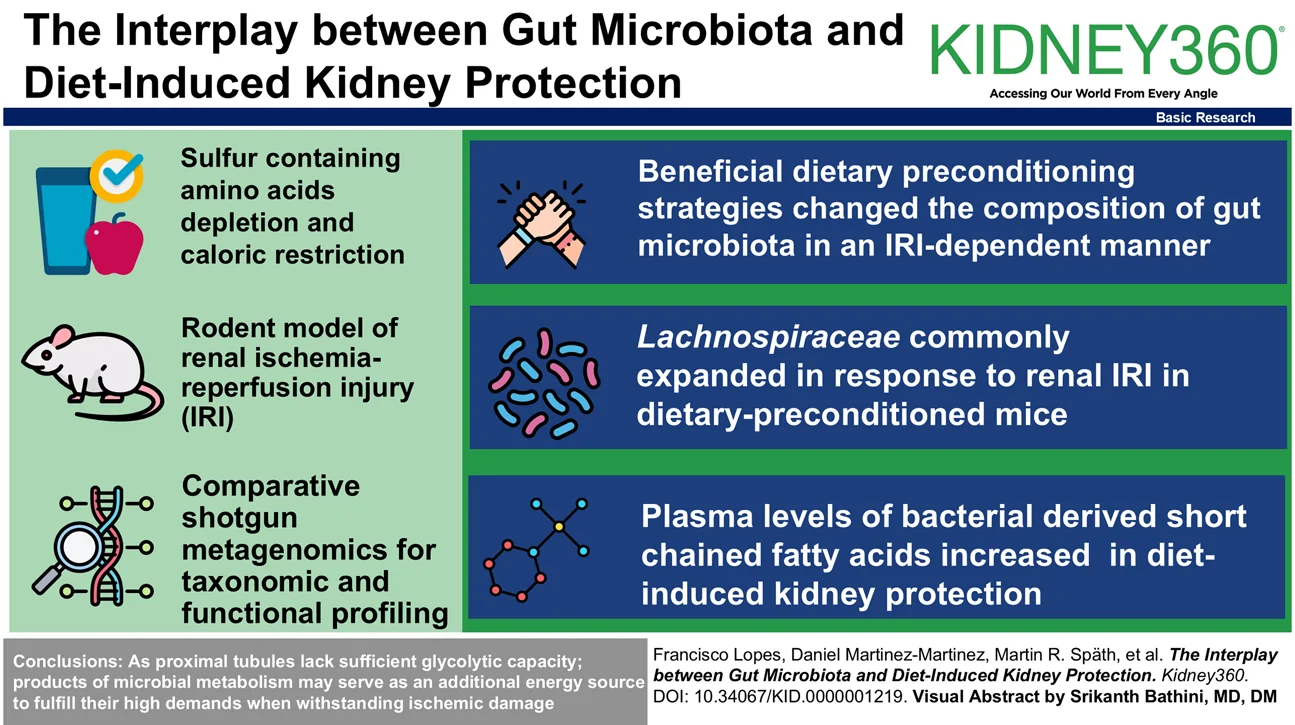

## Introduction

AKI is one of the most common disorders in the clinic feared because of its substantial morbidity and mortality.^1,2^ Despite this considerable burden, effective therapeutic approaches are lacking.^3^ Interestingly, different dietary interventions show an immense potential in preventing kidney injury from various damaging stimuli in rodents, including our own work.^4–6^ In this context, preconditioning with a fasting-mimicking diet (FMD), a diet depleted in the sulfur-containing amino acids (SR), or caloric restriction (CR) led to a significantly improved survival after AKI and strongly protected kidney function from ischemic damage.^4^

However, the limited understanding of the underlying mechanisms is hindering the translation of this immense potential to the clinical setting.^7–9^ Recent findings highlight a central role of gut microbiota in kidney health and disease.^10–12^ While contributing to mucosal immunity and nutrient metabolism in the intestine, gut microbiota preserve the homeostasis in the gastrointestinal tract.^13,14^ Promising modulators are microbiota-derived metabolites, including short-chain fatty acids (SCFA), produced by anaerobic gut microbiota through saccharolytic fermentation of dietary fibers. Importantly, SCFAs reach the bloodstream and, therefore, promise to unravel the connection of diet-induced changes of the gut microbiota, the host metabolome, and diet-induced kidney protection.^13,15–17^

In this study, we aim to delineate the diet-induced effect of different protective dietary regimens on gut microbiota in a rodent model of ischemia–reperfusion injury (IRI)-induced AKI. To increase the likelihood of identifying microbiota-associated mechanisms that generalize beyond a single intervention, we tested three independently protective dietary regimens in parallel (FMD, SR, and CR) and focused on convergent taxonomic/functional signatures shared across protective diets. In aggregate, this work suggests a potential role for the gut microbiota in the regulation of IRI-induced AKI.

## Methods

### Dietary Preconditioning Protocols in Rodents

Dietary preconditioning was performed as previously described.^4^ Briefly, male C57BL6N mice at 6 weeks of age were obtained from Janvier Labs (Paris, France). Upon arrival, they were directly maintained individually in an identical specific pathogen-free environment with 12/12-hour light/dark cycle and constant temperature and humidity. Unless on specific dietary regimens, mice were fed *ad libitum* (AL) with their regular chow (V155-330 ssniff GmbH, Soest, Germany) and had AL access to water. At 8 weeks of age, the mice were allocated to their specific dietary regimen in a randomized manner, and with start of the respective dietary regimen, they were transferred to a fresh cage. The experimental dietary regimens FMD, SR, and CR were performed in parallel for 14 days before renal IRI. The FMD regimens consisted of intermittent fasting and was performed as previously described.^4,18^ Briefly, energy uptake was reduced to 7.67 kJ/g on day 1 (S5159-E736, ssniff GmbH, Soest, Germany) and 1.48 kJ/g on day 2–3 (S5159-E738, ssniff GmbH, Soest, Germany) of intermittent fasting. Specific contents of rodent FMD are summarized in Supplemental Table 1. On day 4–7 of FMD, mice had AL access to their standard chow (Supplemental Table 2). The FMD regimen was performed twice before renal IRI.

The daily uptake of SR was fully restricted in a diet without any methionine and cysteine (SR, S5159-E720, ssniff GmbH, Soest, Germany). SR was supplied AL for 14 days before IRI and was replenished weekly.

In CR animals, the energy uptake was reduced to 70% of their regular intake (3 g/d) using a restricted amount of regular chow supplied in the morning hours (09:00–11:00 am) every day. CR was performed for 14 days before renal IRI, and CR animals were not fed on the day of surgery. When adhering to the different experimental diets, mice had AL access to water.

### Renal IRI Model

AKI was induced using a warm renal IRI model as previously described.^4^ Briefly, mice were anesthetized with intraperitoneal administered ketamine (Zoetis Inc. Parsippany, NJ)/xylazine (Bayer AG, Leverkusen, Germany). To maintain a stable body temperature during surgery, a thermometer was inserted rectally, and mice were placed on a temperature-controlled heating pad (Havard apparatus, Holliston, MA). After midline laparotomy, right-sided nephrectomy was performed followed by the clamping of the left renal pedicle for 40 minutes using two atraumatic microvascular clamps to induce renal IRI. After verification of the kidney color change upon ischemia, the abdomen was covered with a compress soaked in 0.9% saline solution. Once the microvascular clamps were removed, restoration of renal blood flow was confirmed visually. At the end of rodent surgery, the abdomen was closed in two layers and the mice were transferred into a fresh cage. After surgery, both the dietary preconditioned and nonpreconditioned AL fed mice had AL access to the standard chow (Supplemental Table 2). For analgesia, the mice received buprenorphine (Indivior Europe Limited, Dublin, Ireland), in a dosage of 0.1 mg/kg per body weight injected subcutaneously twice daily, in addition to 0.009 mg/ml buprenorphine administered in drinking water. To minimize suffering and distress of the mice, a government-approved recovery score with *a priori* defined humane end points was assessed twice daily after renal IRI (Supplemental Table 3).

Sham animals underwent right-sided nephrectomy without clamping of the vascular pedicle of the contralateral kidney and served as an experimental control following the current consensus recommendations of the International Society of Nephrology for animal studies.^19^ Thus, this sham-with-nephrectomy control accounts for anesthesia, laparotomy, and nephrectomy-associated systemic effects, thereby enabling separation of surgery-related changes from ischemia-specific effects. Mice were euthanized at baseline before IRI as well as 24 or 72 hours after reperfusion. Therefore, mice were anesthetized with intraperitoneal ketamine/xylazine injection, and final blood withdrawal was performed by puncture of the right ventricle. Sham 0 hour and nondietary preconditioned AL fed baseline animals (AL 0 hour) represent the same biologic condition (standard chow, pre-IRI baseline). Consistent with 3R principles (replacement, reduction, and refinement) and to reduce animal numbers, sham 0 hour animals were not duplicated.^20^

Murine stool samples were collected from the animal's colon immediately after the mice were euthanized and directly snap-frozen in liquid nitrogen and stored at −80°C until further use. To quantify kidney function upon renal IRI, serum creatinine and urea levels were measured in a blinded manner in the central laboratory of the University Hospital Cologne using a Cobas C 702 (Roche Diagnostics GmbH, Penzberg and Mannheim, Germany).

Finally, 2-*µ*m-thick slices of formalin-fixed, paraffin-embedded tissue were stained with periodic acid–Schiff to examine tubular cell damage. Images were obtained on a slide scanner SCN4000 (Leica, Wetzlar, Germany) at 20× magnification. A composite histopathologic damage score containing the four items vacuolization, epithelial flattening, loss of brush borders of nuclei, and tubular necrosis was assessed in a blinded and semiquantitative manner by an experienced nephropathologist (H. Göbel).^21,22^ Each of these four items was graded from 0 to 4 representing the affected area (1: 0%–25%, 2: 25%–50%, 3: 50%–75%, 4: 75%–100%), and their scores were summed.

All rodent experiments were approved by the state authorities of North-Rhine Westphalia, Germany (Landesamt für Verbraucherschutz und Ernährung NRW: AZ: 2019.A345). We did not observe any expected or unexpected adverse events during the conduct of the animal study.

### Taxonomic Profiling Using Shotgun Metagenomics

For taxonomic composition of the murine microbiome in the context of the dietary preconditioning strategies and renal IRI, shotgun metagenomics was used. Total DNA extraction, detection, and shotgun metagenomic sequencing were performed on the DNA nanoball sequencing platform (BGI, Shenzhen, China). Briefly, DNA molecules were fragmented, and adapters were ligated to these fragments, generating libraries containing single-strand DNA circles ligated to an adapter. After PCR amplification, the single-strand circular DNA library was sequenced on the DNA nanoball sequencing platform as previously described.^23,24^ Low-quality reads and host reads were removed.^25^ Thus, 12 stool samples were not sequenced.

High-quality reads of the remaining 72 stool samples were aligned to the sequences of the genome taxonomy database references (Oct 22 database) using the MetaPhlAn4 for metagenomic taxonomic profiling, and 401 species-level taxa were identified.^26^ Ecologic analysis was performed using the *R* package microeco (v.1.3.0). *α*-Diversity (within-sample diversity) was calculated using Shannon index depending on the gene abundance. *β*-Diversity (between-sample diversity) was estimated by Bray–Curtis dissimilarity and represented with a principal coordinates analysis, statistical significance was determined with the permutational multivariate  analysis of variance (PERMANOVA; adonis2), and pairwise PERMANOVA comparisons were performed with multiple-testing correction.

### Functional Profiling

Shotgun metagenomic sequencing data from murine fecal samples were first processed for quality control and adapter removal using the short oligonucleotide analysis package.^25^ Functional annotation of metagenomic reads was performed using HUMAnN v3. Nucleotide-level alignment steps were bypassed to reduce computational time, enabled by setting bypass-prescreen, bypass-nucleotide index, and bypass-nucleotide search. The output files, including gene families and pathway abundance, were postprocessed to extract gene ontology (GO) and Kyoto Encyclopedia of Genes and Genomes Orthology terms using custom regrouping tables (map_go_uniref90.txt, map_ko_uniref90.txt) downloaded through the human_databases—download utility_mapping full command. To account for differences in sequencing depth across samples, read counts were computed directly from the original fq.gz files. The final output abundance tables were normalized by dividing each term's abundance by the total number of reads in the corresponding sample, yielding abundance values scaled by true sequencing depth.

### Transcriptomic Analysis

For transcriptomics analysis existing datasets of our working group generated from snap-frozen kidneys of male CR preconditioned and nonpreconditioned AL fed control C57BL6N mice undergoing renal IRI, as described earlier, were used.^27^ Briefly, bulk transcriptome datasets were generated using the Stranded Total RNA Prep with Ribo-Zero Plus on the TruSeq Total RNA platform (Ilumina Inc. San Diego, CA) on a NovaSeq 6000 sequencing platform. Bulk RNA-seq expression was plotted using the heatmap () function from the ComplexHeatmap *R* package (v2.22.0). Before plotting, expression values were z-score scaled per gene (row-wise). GO gene sets of interest were derived from org.Mm.eg.db (v 3.20.0), and for each gene set, a heatmap was produced with genes clustered using hierarchical clustering on rows (Euclidean distance).

### Sample Preparation for Proteomic Analysis Using Liquid Chromatography with Tandem Mass Spectrometry

The renal proteome was established using nano-liquid chromatography and mass spectrometry on a Q Exactive Plus Orbitrap mass spectrometer (in data-dependent acquisition mode) coupled to an EASY nLC 1000 (both Thermo Fisher Scientific, Waltham, MA) as previously described.^4^ Briefly, peptides were chromatographically separated at a constant flow rate of 250 nl/min using the following gradient: 3%–4% solvent B (0.1% formic acid in 80% acetonitrile) within 1.0 minutes, 4%–27% solvent B within 119.0 minutes, 27%–50% solvent B within 19.0 minutes, and 50%–95% solvent B within 1.0 minutes, followed by washing and column equilibration. The mass spectrometry (MS) 1 survey scan was acquired from 300 to 1750 m/z at a resolution of 70,000. Product ions were detected in Orbitrap at a resolution of 17,500. Precursors were dynamically excluded for 30.0 seconds.

### SCFA Quantification

Levels of acetate and propionate in mouse plasma were determined by anion-exchange chromatography coupled to electrospray ionization high-resolution mass spectrometry: To 400 *µ*l of ice-cold methanol, 100 *µ*l of plasma and 20 *µ*l of a mixture of isotope-labeled internal standards in Milli-Q water (100 *µ*M Natriumacetat-^13^C_2_, d_3_ and 100 *µ*M Propionsäure-d_6_, both Sigma–Aldrich, St. Louis, MO) were added. The samples were vortexed for 20 seconds and incubated at −70°C for 30 minutes. The samples were then extracted using a precooled ThermoMixer (Eppendorf, Hamburg, Germany) at 4°C and at 3.000 rpm for 15 minutes. After centrifugation (16,100 relative centrifugal force (RCF), 20 minutes, 4°C), the supernatants were removed, transferred to new tubes, vortexed for 20 seconds, and again incubated at −70°C for 30 minutes. After centrifugation (16,100 RCF, 20 minutes, 4°C), the supernatants were removed, transferred to new tubes, and dried under a stream of nitrogen. The dried extracts were re-solved in 100 *µ*l of Milli-*Q* water. After mixing and centrifugation (16,100 RCF, 20 minutes, 4°C), 80 *µ*l of the supernatants were transferred to autoinjector vials and immediately measured. Ion chromatography coupled to high-resolution MS analysis was performed using a dionex integrion reagent-free ion chromatography system (Thermo Scientific, Waltham, MA) equipped with a Dionex IonPac AS11-HC column (2×250 mm, 4 *μ*m particle size, Thermo Scientific, Waltham, MA) and a Dionex IonPac AG11-HC guard column (2×50 mm, 4 *μ*m, Thermo Scientific, Waltham, MA) and coupled to a *Q* Exactive HF quadrupole-Orbitrap mass spectrometer (Thermo Scientific, Waltham, MA). Five *µ*l of the sample were injected using a Dionex AS-AP at 5°C. The ion chromatography was operated at a flow rate of 0.38 ml/min with a potassium hydroxide gradient that was produced by an eluent generator with a potassium hydroxide cartridge and Milli-*Q* water. The gradient started with 10 mM potassium hydroxide over 3 minutes, 10−50 mM from 3 to 12 minutes, 50−100 mM from 12 to 19 minutes, held at 100 mM from 19 to 25 minutes, and re-equilibrated at 10 mM for 3 minutes. A Dionex ADRS 600, 2-mm suppressor was operated with 95 mA, and methanol/water 95:5 (v/v) was used to produce a makeup flow at a flow rate of 0.15 ml/min.^28^ The mass spectrometer was operated in the negative ion mode. Full MS scans in the range of 50–120 m/z were acquired with a resolution of 15,000, automatic gain control target value of 3×10^6^, and maximum injection time of 240 ms. Spectrum data were collected in the profile mode. The electrospray ionization source was operated with flow rates for sheath gas, auxiliary gas, and sweep gas of 30, 14, and 3, respectively. The spray voltage setting was 2.75 kV, capillary temperature 230°C, S-lens RF level 60, and auxiliary gas heater temperature 380°C. The exact m/z traces of acetate and propionate and the isotope-labeled internal standards were extracted and integrated using TraceFinder 5.1 software (Thermo Scientific, Waltham, MA). Endogenous acetate and propionate were quantified on the basis of external calibration curves that were calculated from ion chromatography-mass spectrometry measurements of diluted synthetic acetate and propionate standards (Sigma-Aldrich, St. Louis, MO). Linearity and correlation coefficients of the standard curves were obtained by linear regression analysis.

### Statistical Analyses

All statistical analyses were performed using *R*. Unless stated otherwise, tests were two-sided and *P* values < 0.05 were considered significant. Where multiple hypotheses were tested, *P* values were adjusted using the Benjamini‑Hochberg false discovery rate procedure.

Transcriptomics differential gene expression was tested using a voom-transformed linear model fitted with lmFit, specified contrasts were applied with contrasts.fit, and SEMs were moderated using empirical Bayes (eBayes). The design matrix was specified as model.matrix (approximately 0+group).

For proteomics, differential protein abundance was tested using limma by fitting a linear model with lmFit, applying specified contrasts using contrasts.fit, and empirical Bayes moderation using eBayes, with the design matrix specified as model.matrix (approximately 0+group).

Other continuous outcomes (creatinine, urea, weight, and recovery score), diet effects, and IRI effects were evaluated using Welch two-sample *t* tests; effect sizes are reported as mean differences between groups. Diet×IRI interaction effects were evaluated using a linear model including an interaction term (y approximately diet×time), with interaction effect sizes interpreted as difference in differences.

Ordinal histology/score outcomes (histopathologic damage score, its respective items vacuolization, epithelial flattening, loss of brush borders, loss of nuclei, and necrosis), in addition to diet effects and IRI effects, were evaluated using Wilcoxon rank-sum tests; effect sizes are reported as median differences between groups. Diet×IRI interaction effects were evaluated using a rank-based linear model (rank [y] approximately diet×time), with interaction effect sizes interpreted as difference in differences of medians.

Microbiome diversity beta diversity was assessed using Bray–Curtis dissimilarities and tested by PERMANOVA (adonis2), including pairwise PERMANOVA comparisons with multiple-testing correction. Alpha diversity was analyzed using two-way ANOVA (diet×condition) followed by Tukey *post hoc* multiple-comparisons testing.

Rodent experiments were not blinded because of the different diets. The histologic damage score, SCFA analyses, as well as serum creatinine and serum urea measurements were performed blinded. Omics analyses were conducted unblinded. To reduce potential cofounders, mice were single-caged 2 weeks before dietary interventions with the respective cages located in the same room. Animals treated with the different dietary regimens underwent renal IRI in parallel to further limit batch effects with the same staff performing rodent surgeries. Regarding the omics data, all samples were sequenced parallelly to minimize batch effects. Sample size was chosen on the basis of experience with previous studies using renal IRI and dietary interventions in rodents, and no power analysis was conducted to determine samples sizes.^4^

### Functional Enrichment Analysis

Significant differentially expressed genes from each comparison were used for GO biologic process enrichment analyses conducted with compareCluster from the clusterProfiler package (v4.14.6) and org.Mm.eg.db annotation database, using the default parameters. To facilitate interpretation, log2 fold-change values were linked to enriched GO terms. For each comparison, for every enriched term, an average log2 fold-change across the associated genes was computed and added to the enrichment result. Enrichment results were visualized using dotplot from clusterProfiler, with showCategory=5 to display the top five enriched categories of each and across the four different comparisons. Dot size represented adjusted *P* values, while dot color encoded the mean log2 fold-change (blue=negative, red=positive).

## Results

### Beneficial Dietary Preconditioning Regimens Protected the Kidney from Ischemic Damage

In a systematic analysis, we quantified the protective effects of three dietary preconditioning—FMD, a diet fully depleted in SR, and CR (66% of the average daily intake)—with all dietary regimens performed for 14 days before renal IRI.^4^ AL fed animals served as negative controls and sham animals undergoing right-sided nephrectomy without clamping of the vascular pedicle of the left kidney served as additional controls for the surgical setting. Mice were euthanized before renal IRI and at 24 and 72 hours after reperfusion to quantify kidney function and collect stool and kidney samples for further analyses (Figure 1A).

**Figure 1:**
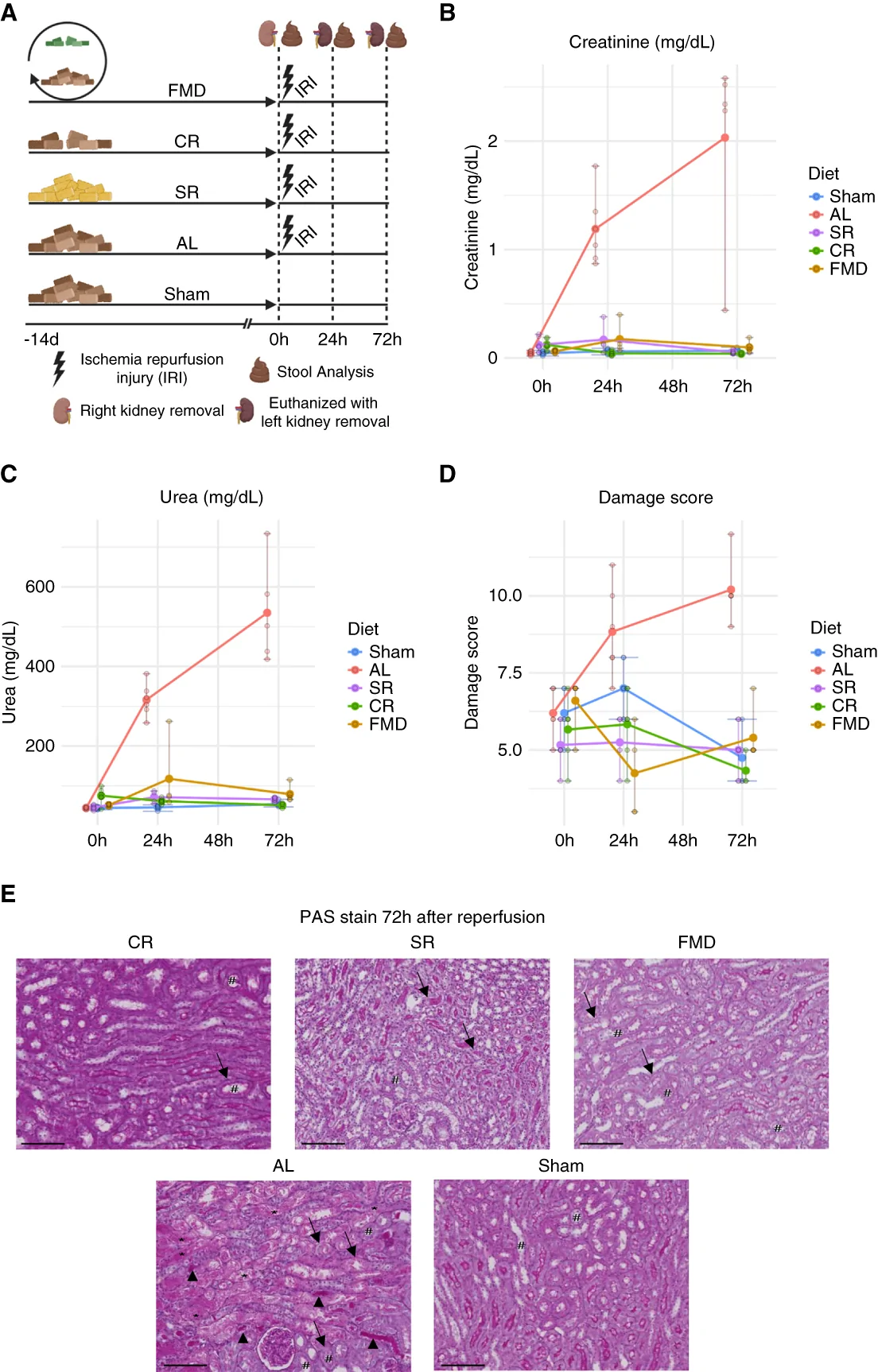
**Dietary interventions protect the kidney from ischemic damage in a rodent model of renal IRI.** (A) Experimental design depicting dietary preconditioning modes. Nonpreconditioned AL fed animals served as negative control. Sham animals underwent right-sided nephrectomy without clamping of the left renal pedicle and did not develop AKI. (B and C) Plasma (B) creatinine and (C) urea quantifications revealed near-to-normal kidney function in mice preconditioned by FMD, SR, or CR examined in our rodent model of renal IRI. (D) A semiquantitative histologic damage score showed little to no tubular damage in dietary preconditioned animals in response to ischemic kidney damage. (B–D) Datapoints represent individual mice; lines represent group means. *n* = 4–6 mice per group per time point. Two-way ANOVA (diet×time) with Tukey multiple-comparisons test; adjusted *P* values and effect sizes summarized in Supplemental Figure 2C. (E) Respective PAS stains of kidneys obtained from dietary preconditioned, AL fed control, and sham animals collected 72 hours after reperfusion. *n* = 4–6 mice per group per time point. Arrows indicate nuclei loss, arrowheads tubular casts, # epithelial flattening, and * denuded tubuli with luminar debris. Bar=100 *µ*m. Figure 1A was made with bioRender.com. AL, *ad libitum*; CR, caloric restriction; FMD, fasting-mimicking diet; IRI, ischemia‑reperfusion injury; PAS, period acid–Schiff; SR, a diet depleted in the sulfur-containing amino acids.

Dietary preconditioning with FMD, SR, and CR efficiently protected the kidney from ischemic damage, in line with previous reports.^4^ In mice preconditioned with these beneficial dietary protocols undergoing renal IRI, both serum creatinine and serum urea did not differ from sham-operated animals, whereas kidney function in AL fed control animals was significantly impaired (Figure 1, B and C).^4^

To examine the cellular damage in the renal tubular epithelium after IRI, we used an established semiquantitative histologic damage score containing the quantification of epithelial flattening, loss of brush borders, loss of nuclei, necrosis, and vacuolization conducted in a blinded manner by an experienced nephropathologist.^21,22^ In this context, we detected significantly less acute tubular damage in dietary preconditioned animals as compared with nonpreconditioned AL fed control mice quantified both 24 and 72 hours after reperfusion that was primarily driven by reduced tubular necrosis (Figure 1, D and E and Supplemental Figure 1). To sum up, we were able to recapitulate significant health benefits—in line with previous reports—in dietary preconditioned mice as compared with AL fed control mice quantified using a government-approved recovery score consisting of the items weight loss, appearance, and activity (Supplemental Figure 2 and Supplemental Table 3).^4^

### Beneficial Dietary Preconditioning Strategies Changed the Composition of Gut Microbiota in an IRI-Dependent Manner

To assess diet-induced changes in gut microbiota composition, we performed shotgun metagenomic sequencing of stool samples collected from C57BL/6N mice in a rodent model of renal IRI after dietary preconditioning and respective controls. In this context, we calculated *α*-diversity (within-sample diversity) using the Shannon index and *β*-diversity (between-sample diversity) using the Bray–Curtis dissimilarity (Figure 2, A–C). Strikingly, we detected a strong change in microbial diversity in response to SR in an IRI-dependent manner. Although CR did not result in a significant change in *α*-diversity, we detected a significant CR-induced separation in *β*-diversity. FMD did not cause a significant change in microbial diversity compared with the AL fed control animals, neither at 24 hours nor at 72 hours after reperfusion (Figure 2, A–C). Because FMD mice were back on standard chow for 4 days before IRI as  part of the periodic fasting regimen, the absence of detectable microbiota differences is consistent with reports that major compositional changes can occur within 48-96 hours after dietary changes (Supplemental Tables 1 and 2).^4,29^ *β*-diversity differences were formally tested by PERMANOVA (Bray–Curtis; adonis2), including pairwise PERMANOVA comparisons with multiple-testing correction. Shannon *α*-diversity was analyzed by two-way ANOVA (diet×condition) with Tukey *post hoc* test. The results are indicated by grouped letters in Figure 2A.

**Figure 2:**
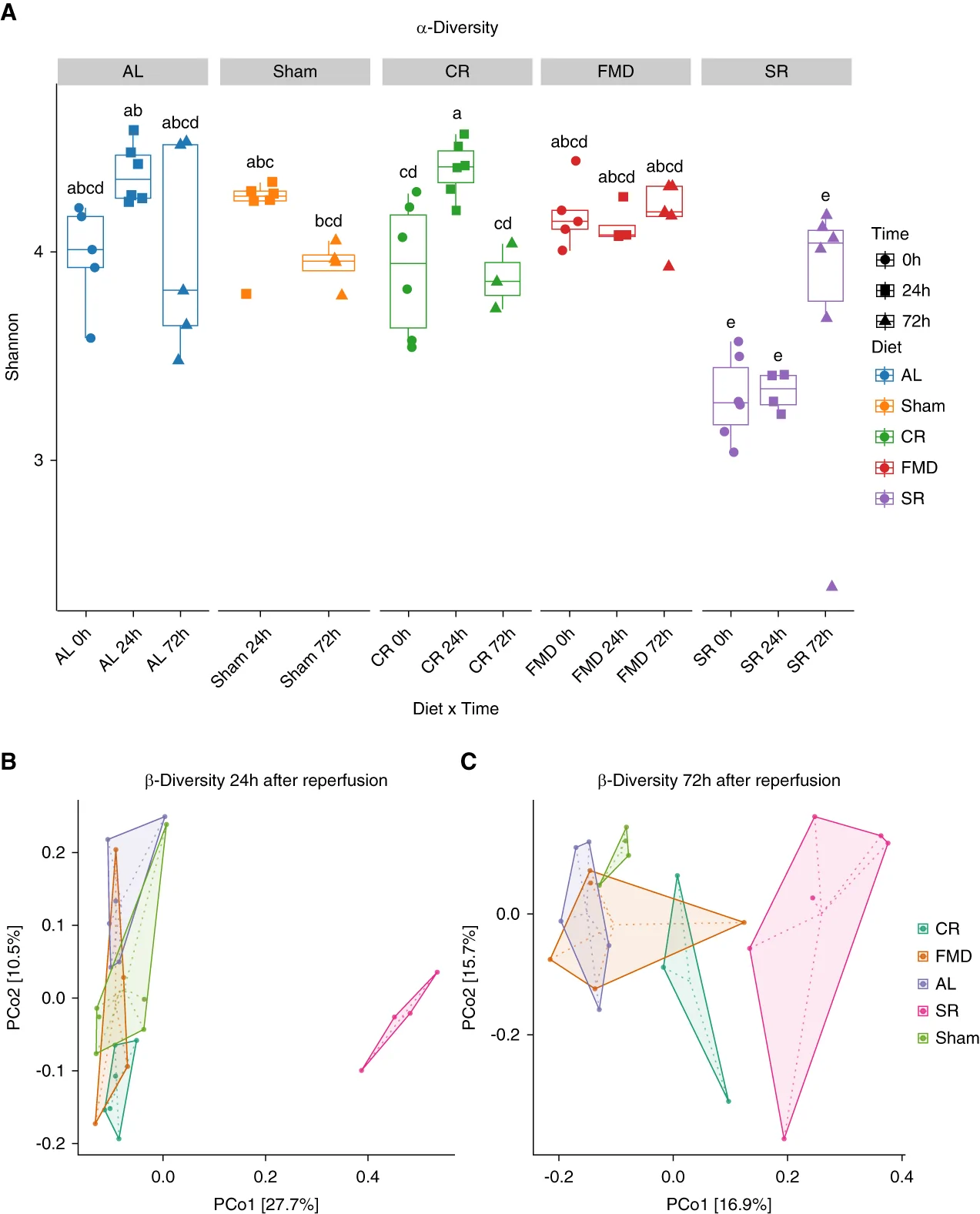
**CR/SR overlappingly change the composition of gut microbiota in an IRI-dependent manner.** (A) *α*-diversity (within-sample diversity) as calculated using the Shannon index of fecal shotgun metagenomes across diet×time groups (0, 24, and 72 hours). Boxplots show median and IQR; points represent individual mice. Letters denote statistically indistinguishable groups on the basis of *post hoc* testing. (B and C) *β*-diversity (between-sample diversity) estimated by Bray–Curtis dissimilarity represented as with a principal coordinate analysis (B) 24 hours and (C) 72 hours after reperfusion. Polygons indicate group convex hulls. *n* = 4–6 mice per group per time point. Statistics: (A) two-way ANOVA (diet×time) followed by Tukey *post hoc* test (compact letter display). (B and C) PERMANOVA on Bray–Curtis distances with, followed by pairwise PERMANOVA with false discovery rate correction. IQR, interquartile range; PCo, prinicipal coordinate; PERMANOVA, permutational multivariate  analysis of variance.

### CR/SR Commonly Increased the Abundance of *Lachnospiraceae* in an IRI-Dependent Manner

Using ternary plots to investigate the role of dietary interventions over time before and after ischemic insult, we observed that the diet-induced changes in microbial composition before IRI were primarily driven by an increase of *Bacteroidota* with a simultaneous decrease of *Firmicutes*. In response to the ischemic insult, we detected, in turn, a common rise in the *Firmicutes* fraction in the SR and CR groups, which was not present among AL fed control animals (Figure 3A). This diet-induced shared change of bacterial phylum was commonly driven by the increased abundance of *Lachnospiraceae*, while *Muribaculaceae* decreased in an IRI-dependent manner (Figure 3B). *Lachnospiraceae* are a main producer of microbially derived SCFAs acetate, butyrate, and propionate.^30,31^ In line with our taxonomic profiling, our functional term “abundance analysis” revealed a rise in SCFA metabolic process among SR/CR preconditioned animals in response to renal IRI that did not differ from sham animals (Figure 4A). Of note, SCFAs are, in turn, known to prevent kidney dysfunction from ischemic damage in rodent models of renal IRI.^15^

**Figure 3:**
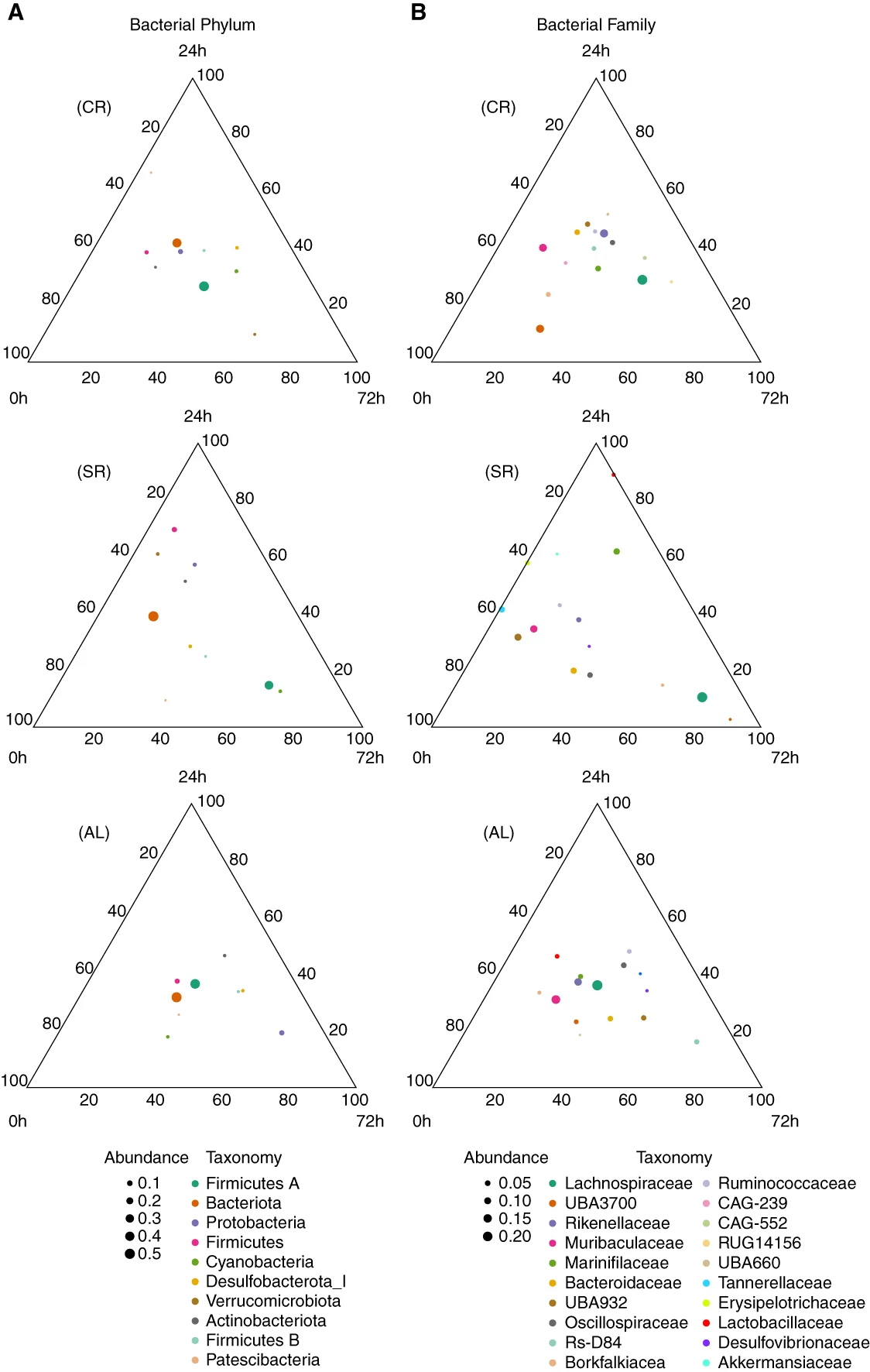
**Ternary plots reveal a common CR/SR-induced change among *Bacteroidota* and *Firmicutes* with a central role of abundant *Lachnospiraceae* in response to renal IRI.** Ternary plots display the relative contribution of each time point to the overall mean abundance of taxa within each diet group (corners: 0 hours baseline, 24 hours after reperfusion, and 72 hours after reperfusion). (A) Phylum-level composition and (B) family-level composition for CR, SR, and AL groups. Each point represents one taxon, point size encodes mean relative abundance (see scale), and color denotes taxonomy. Underlying fecal shotgun metagenomic profiles were generated from *n* = 4–6 mice per diet per time point.

**Figure 4:**
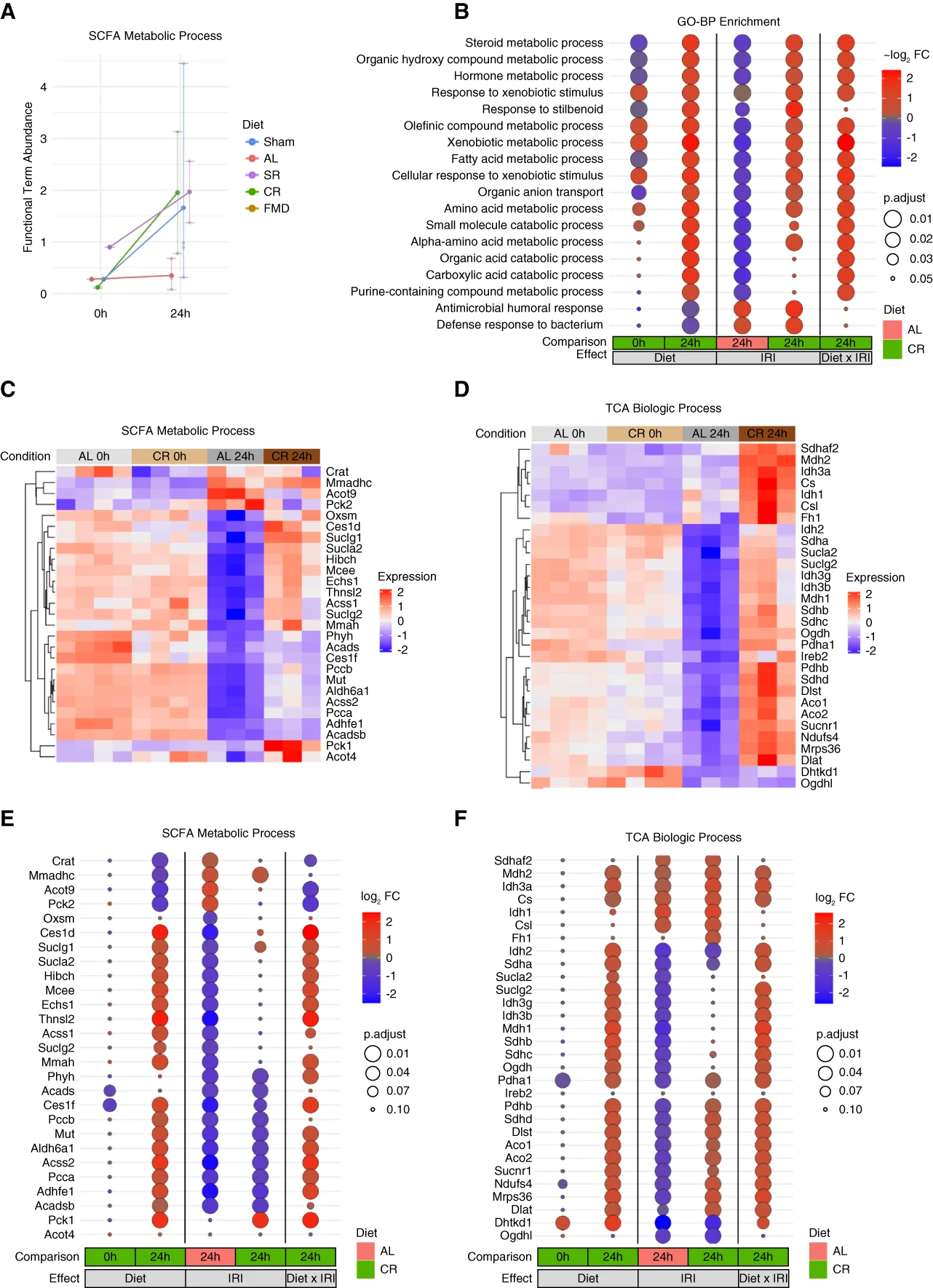
**Functional microbiome signatures converge on SCFA metabolism and align with renal metabolic reprogramming in protected mice.** (A) Abundance of the GO functional term “SCFA metabolic process” inferred from shotgun metagenomics at 0 and 24 hours across AL, CR, SR, and sham groups. Functional term abundance analyses revealed an overlapping increase in SCFA metabolic process in CR/SR preconditioned mice in response to ischemic damage. (B) GO-BP enrichment dot plot across the RNAseq contrasts used in this study (diet at 0 hours, diet at 24 hours, IRI effect at 24 hours within diet, and the diet×IRI interaction at 24 hours). Terms shown are the top five GO-BP categories selected by clusterProfiler's default dot plot ranking from the significant enrichment results; for those same terms, statistics are displayed for all contrasts (including contrasts where the term is NS). Dot color represents the mean core gene log2 fold-change for the term (mean log2 fold-change of genes annotated to the term), and dot size represents the BH-adjusted enrichment *P* value. The unsupervised enrichment analysis detected fatty acid metabolism to be enriched by CR at 0 hours (CR 0 hours) and at 24 hours after IRI (CR 24 hours) as compared with respective kidneys obtained in AL fed control animals. Fatty acid metabolism was further upregulated in kidneys of CR preconditioned animals in response to renal IRI (IRI CR) when compared with the kidney of the same animal collected during surgery after CR but before IRI, whereas renal IRI resulted in a downregulation of fatty acid metabolism in AL fed control animals (IRI AL). (C and D) Heatmaps of *z*-score‑scaled expression (row-wise) for genes in the “SCFA metabolic process” (C) and the “TCA BP” gene sets across AL 0 hours, CR 0 hours, AL 24 hours, and CR 24 hours in kidney RNAseq. (E and F) Gene-level dot plots showing RNAseq differential expression results for (E) SCFA metabolism-related genes and (F) TCA cycle-related genes across the same set of contrasts (diet at 0 hours, diet at 24 hours, IRI effect at 24 hours within diet, and the diet×IRI interaction at 24 hours). Dot color indicates log2 fold-change for each gene in the indicated contrast; dot size indicates the BH-adjusted *P* value from the corresponding test. Differential expression was computed with limma using voom-transformed counts (empirical Bayes-moderated statistics). BP, biologic process; GO, gene ontology; GO-BP, gene ontology enrichment analysis of biologic processes; IRI CR, ischemic kidney damage under CR feeding; SCFA, short-chain fatty acid; TCA, tricarboxylic acid.

### Comparative Kidney RNAseq Analyses Point toward a Central Role of Short-Chain Fatty Acid Metabolism in Diet-Induced Kidney Protection

To unravel the interplay between gut microbiota and kidney protection, we examined the renal metabolic response using comparative transcriptomics. As a starting point, we performed a gene ontology enrichment analysis of biologic processes with fatty acid metabolism being enriched by CR before (CR 0 hour) and after (CR 24 hours) ischemic kidney damage and in response to ischemic kidney damage under CR feeding (Figure 4B). By contrast, renal IRI lead to a downregulation of fatty acid metabolism in AL fed control mice.

In this context, the acyl-CoA synthetase short-chain family members 1 and 2 (Acss1/Acss2) were upregulated in CR preconditioned animals in response to ischemic kidney damage as compared with AL fed control animals (Figure 4C). Acss1 and Acss2 generate acetyl-CoA as well as propionyl-CoA from acetate and propionate fueling bacterial-derived acetate and propionate into the tricarboxylic acid (TCA). Consistent with this observation, we identified a strong upregulation of phosphoenolpyruvate carboxykinase 1 forming phosphoenolpyruvate from oxaloacetate and bypassing acetyl-CoA in CR-treated animals in response to ischemia. Of note, we were able to recapitulate this finding on the protein levels in CR-preconditioned animals undergoing renal IRI using kidney bulk proteomics (Supplemental Figure 3). By contrast, we did not detect a significant change in Acyl-CoA dehydrogenase levels involved in the production of butyryl-CoA from butyrate (Figure 4C).

Propionate bypasses the first four TCA enzymes when being processed by propionyl-CoA to succinyl-CoA in the presence of vitamin B12 or to acetyl-CoA in its absence and, thus, causes a shift in the TCA when entering. Of note, the propionate‑to‑propionyl-CoA and propionyl-CoA‑to‑succinyl-CoA converting enzymes such as methylmalonyl-CoA epimerase, succinyl-CoA ligase subunit *β*, and succinate-CoA ligase ADP-forming subunit *β* were found upregulated by CR in an IRI-dependent manner, indicating an overall increase in propionate metabolism through the vitamin B12‑dependent pathway (Figure 4, C and D).

### CR/SR Commonly Increases Plasma SCFA Levels

To corroborate these functional predictions, we quantified acetate and propionate plasma by ion chromatography coupled to high-resolution MS. In this context, we quantified significantly higher acetate plasma levels in SR-treated animals compared with AL, whereas we detected a trend toward elevated acetate plasma levels in response to CR that, however, failed to reach significance shortly (*P*=0.14) (Figure 5A). More importantly, adhering to SR and CR resulted in significantly increased propionate plasma levels (Figure 5B). On the other hand, and in line with our shotgun metagenomics results, FMD increased neither acetate nor propionate plasma levels.

**Figure 5:**
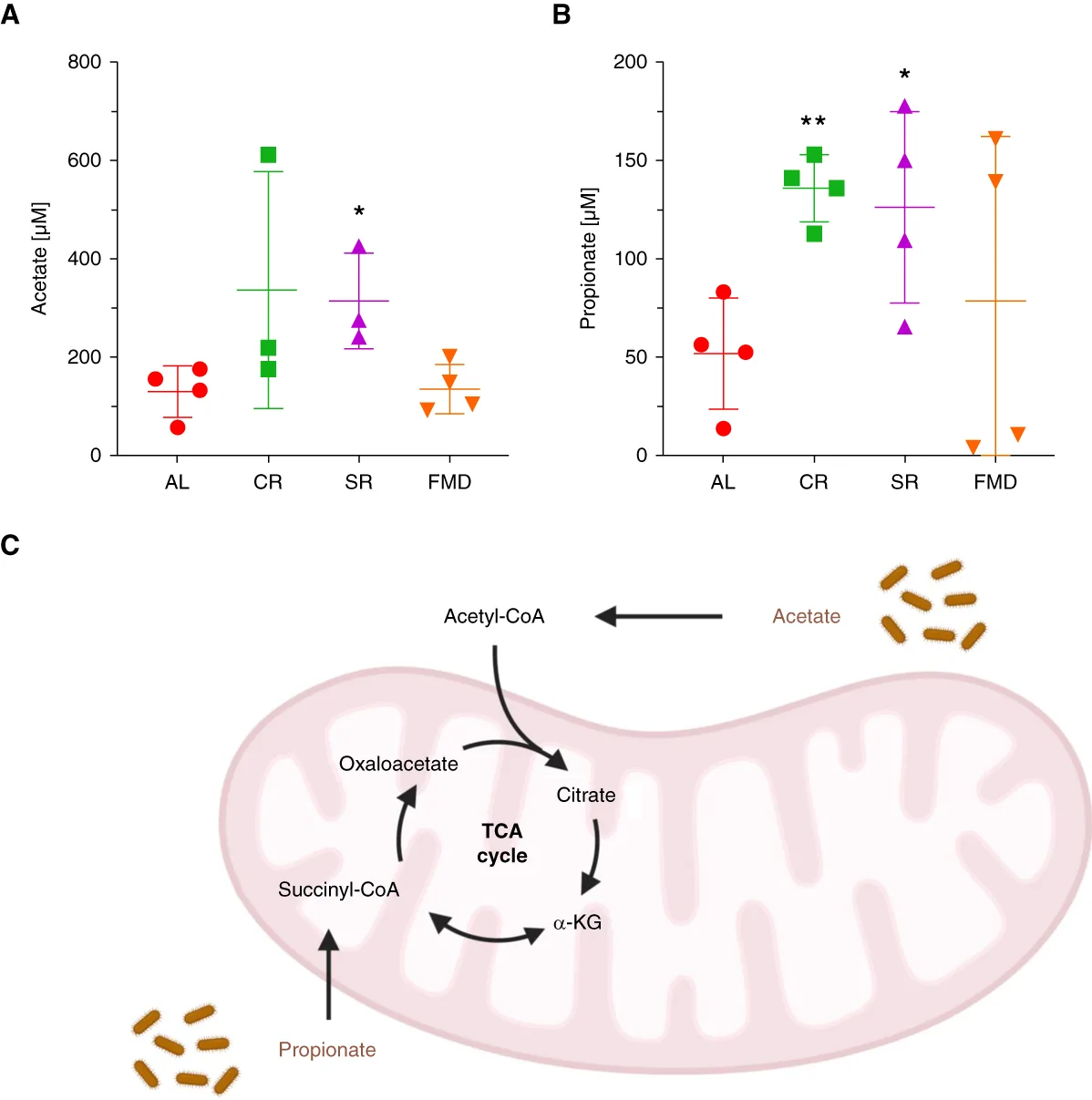
**The microbially generated SCFA acetate and propionate may serve as an additional energy source for the kidney to withstand ischemic damage.** (A and B) Plasma (A) acetate and (B) propionate levels in CR, SR, and FMD-treated animals in addition to AL fed mice as detected by IC-HRMS. Mean and SD are given, and each animal is depicted as a single dot. *n* = 3–4 mice per group. Two-tailed *t* tests were used to test for significance as indicated. (C) Conceptual model linking microbiota-derived SCFAs to renal mitochondrial metabolism after ischemic injury. Acetate converts to acetyl-CoA, feeding into the TCA cycle, whereas propionate can contribute anaplerotically by conversion to succinyl-CoA/oxaloacetate. This schematic summarizes a plausible route by which increased SCFA availability in protected diets may support proximal tubular energy demands during recovery from IRI. Figure 5C was created with bioRender.com. IC-HRMS, ion chromatography coupled to high-resolution MS; MS, mass spectrometry.

## Discussion

On one hand, gut microbiota plays an important role in kidney health and disease.^10,15^ On the other hand, dietary interventions are known for their pivotal role in regulating diversity, composition, and function of the gut microbiome.^30^ As diet-induced kidney protection may substantially add to our therapeutic toolkit, we systematically analyzed gut microbial changes induced by beneficial dietary regimens in the context of renal IRI.^4^

In this context, we found the most protective dietary regimens SR and CR to commonly increase the *Bacteroidota*/*Firmicutes* ratio before IRI.^4^ This diet-induced ratio changes among *Bacteroidota* and *Firmicutes* has also been reported to be caused by CR in longevity studies using C57BL/6JN mice resulting in stress resistance and increased lifespan and health span.^32^ Thus, it is striking that even short-term dietary interventions, as reflected by 2 weeks of the SR or CR regimen, were capable of producing this profound change among gut microbiota, as observed in long-term CR studies.^32^ Likewise, a recent report identified short-term CR to decrease levels of *Firmicutes* among gut microbiota of rhesus macaques.^33^ By contrast, high-fat diets and resulting obesity in rodents and humans alter gut microbiome composition in an opposite manner.^30^ This points toward an evolutionary conserved diet-induced change in the composition of gut microbiota, an important prerequisite for translational applicability to humans.

Interestingly, levels of *Lachnospiraceae* strongly increased in the SR and CR preconditioning mice in response to renal IRI. Likewise, this change was also found in diet-induced liver protection in a rodent model of hepatic IRI.^34^ Our functional analysis revealed an overlapping, IRI-dependent enrichment in SCFA metabolic process among gut microbiota of SR and CR preconditioned animals. In this context, *Lachnospiraceae* are reported to be among the primary producers of SCFA that enter the blood stream, and, in turn, we detected elevated acetate and propionate plasma levels in response to beneficial diets.^30,31^ Bulk transcriptomics of kidney in our model further revealed significant changes in a diet-dependent manner. In particular, we found that metabolic genes involved in the use of acetate and propionate, products of microbial metabolism that enter the renal TCA, to be enriched in the context of diet-induced kidney protection (Figure 5C).^15^ Because AKI-susceptible proximal tubules lack sufficient glycolytic capacity, lipid metabolism and the TCA play a central role to fulfill their high energy demands.^35^ Our data shed light on a close interplay between gut microbiota and diet-induced kidney protection calling for further research at the crossroad of microbiology, metabolism, and molecular nephrology. At this point, additional causal tests strengthening our hypothesis such as SCFA supplementation, antibiotic depletion of harmful microbiota, and fecal transfer of protective microbes examined in our rodent model are promising next steps to be taken. In this context, emerging human multiomics efforts in AKI, including the Kidney Precision Medicine Project, provide an additional framework to test whether microbiome-associated metabolites and renal metabolic programs observed are similarly engaged in human AKI phenotypes.^34^ Integrating stool metabolomics/microbiome profiles with kidney biopsy transcriptomics in such cohorts may help to prioritize SCFA-linked pathways as candidate targets in patients.

## Supplementary Material

## Data Availability

Datasets of shotgun metagenomics are uploaded and freely available at the European Nucleotide Archive platform. Proteomics datasets are uploaded and freely available the PRIDE repository. The bulks transcriptomics datasets are available at https://uni.cologne/LAWNQ.

## Declarative Statements

All animal experiments were conducted in accordance with the NIH Guide for the Care and Use of Laboratory Animals or an equivalent standard that meets or exceeds the ethical and welfare requirements outlined in the NIH Guide. All protocols were approved by the appropriate institutional animal care and use committee.

## Supplemental Material

Supplemental Table 1. Content and metabolizable energy of the FMD components used on day 1 and day 2/3.

Supplemental Table 2. Proximate content and metabolizable energy of a diet depleted in the SR and the standard chow used for dietary preconditioning.

Supplemental Table 3. Government-approved recovery score after renal IRI based on weight loss, appearance, and activity.

Supplemental Figure 1. Quantification of the items (A) epithelial flattening, (B) loss of brush borders, (C) loss of nuclei, (D) necrosis, and (E) vacuolization of the used histologic damage score established in a blinded manner in our model of renal IRI.

Supplemental Figure 2. A governmental recovery score after renal IRI using the three items weight loss, activity, and appearance (Supplemental Table 1) was used to quantify health after renal IRI.

Supplemental Figure 3. Recapitulation of diet-induced metabolic reprogramming on the protein level in kidneys of protected mice.

Supplemental References.
