# Supplementary material for: The Interplay between Gut Microbiota and Diet-Induced Kidney Protection
Source: Kidney360. 2026 May 26;7(9):1930–42. doi: 10.34067/KID.0000001219 (PMC13641456; doi:10.34067/KID.0000001219)
Supplement: Supplementary file 1 [file kidney360-7-1930-s001.pdf]

ASN American Society of Nephrology
JASN Journal of the American Society of Nephrology
KIDNEY360 Accessing Our World From Every Angle
CJASN Clinical Journal of the American Society of Nephrology

ASN Journal Disclosure Form

As per ASN journal policy, I have disclosed any financial relationships or commitments I have held in the past 36 months as included below. I have listed my Current Employer below to indicate there is a relationship requiring disclosure. If no relationship exists, my Current Employer is not listed.

T. Benzing reports the following:
Employer: University of Cologne

I understand that the information above will be published within the journal article, if accepted, and that failure to comply and/or to accurately and completely report the potential financial conflicts of interest could lead to the following: 1) Prior to publication, article rejection, or 2) Post-publication, sanctions ranging from, but not limited to, issuing a correction, reporting the inaccurate information to the authors' institution, banning authors from submitting work to ASN journals for varying lengths of time, and/or retraction of the published work.

Name: Thomas Benzing
Manuscript ID: K360-2025-001311R3
Manuscript Title: The interplay between gut microbiota and diet-induced kidney protection.
Date of Completion: May 4, 2026
Disclosure Updated Date: March 25, 2026

ASN Journal Disclosure Form

As per ASN journal policy, I have disclosed any financial relationships or commitments I have held in the past 36 months as included below. I have listed my Current Employer below to indicate there is a relationship requiring disclosure. If no relationship exists, my Current Employer is not listed.

A. Beyer reports the following:
Employer: University of Cologne; and Other Interests or Relationships: Member of the ISCB - International Society for Computational Biology.

I understand that the information above will be published within the journal article, if accepted, and that failure to comply and/or to accurately and completely report the potential financial conflicts of interest could lead to the following: 1) Prior to publication, article rejection, or 2) Post-publication, sanctions ranging from, but not limited to, issuing a correction, reporting the inaccurate information to the authors' institution, banning authors from submitting work to ASN journals for varying lengths of time, and/or retraction of the published work.

Name: Andreas Beyer
Manuscript ID: K360-2025-001311R1
Manuscript Title: The interplay between gut microbiota and diet-induced kidney protection
Date of Completion: March 5, 2026
Disclosure Updated Date: March 5, 2026

ASN American Society of Nephrology
JASN Journal of the American Society of Nephrology
KIDNEY360 Accessing Our World From Every Angle
CJASN Clinical Journal of the American Society of Nephrology

ASN Journal Disclosure Form

As per ASN journal policy, I have disclosed any financial relationships or commitments I have held in the past 36 months as included below. I have listed my Current Employer below to indicate there is a relationship requiring disclosure. If no relationship exists, my Current Employer is not listed.

S. Brodesser has nothing to disclose.

I understand that the information above will be published within the journal article, if accepted, and that failure to comply and/or to accurately and completely report the potential financial conflicts of interest could lead to the following: 1) Prior to publication, article rejection, or 2) Post-publication, sanctions ranging from, but not limited to, issuing a correction, reporting the inaccurate information to the authors' institution, banning authors from submitting work to ASN journals for varying lengths of time, and/or retraction of the published work.

Name: Susanne Brodesser
Manuscript ID: K360-2025-001311R1
Manuscript Title: The interplay between gut microbiota and diet-induced kidney protection
Date of Completion: March 5, 2026
Disclosure Updated Date: March 5, 2026

ASN American Society of Nephrology | JASN Journal of the American Society of Nephrology | KIDNEY360 Accessing Our World From Every Angle | CJASN Clinical Journal of the American Society of Nephrology

ASN Journal Disclosure Form

As per ASN journal policy, I have disclosed any financial relationships or commitments I have held in the past 36 months as included below. I have listed my Current Employer below to indicate there is a relationship requiring disclosure. If no relationship exists, my Current Employer is not listed.

F. Cabreiro reports the following:
Advisory or Leadership Role: Frontiers

I understand that the information above will be published within the journal article, if accepted, and that failure to comply and/or to accurately and completely report the potential financial conflicts of interest could lead to the following: 1) Prior to publication, article rejection, or 2) Post-publication, sanctions ranging from, but not limited to, issuing a correction, reporting the inaccurate information to the authors' institution, banning authors from submitting work to ASN journals for varying lengths of time, and/or retraction of the published work.

Name: Filipe Cabreiro
Manuscript ID: K360-2025-001311R1
Manuscript Title: The interplay between gut microbiota and diet-induced kidney protection
Date of Completion: March 4, 2026
Disclosure Updated Date: March 4, 2026

ASN American Society of Nephrology
JASN Journal of the American Society of Nephrology
KIDNEY360 Accessing Our World From Every Angle
CJASN Clinical Journal of the American Society of Nephrology

ASN Journal Disclosure Form

As per ASN journal policy, I have disclosed any financial relationships or commitments I have held in the past 36 months as included below. I have listed my Current Employer below to indicate there is a relationship requiring disclosure. If no relationship exists, my Current Employer is not listed.

S. Cukoski reports the following:
Employer: University Hospital of Cologne

I understand that the information above will be published within the journal article, if accepted, and that failure to comply and/or to accurately and completely report the potential financial conflicts of interest could lead to the following: 1) Prior to publication, article rejection, or 2) Post-publication, sanctions ranging from, but not limited to, issuing a correction, reporting the inaccurate information to the authors' institution, banning authors from submitting work to ASN journals for varying lengths of time, and/or retraction of the published work.

Name: Sadrija Cukoski
Manuscript ID: K360-2025-001311R3
Manuscript Title: The interplay between gut microbiota and diet-induced kidney protection
Date of Completion: April 29, 2026
Disclosure Updated Date: April 29, 2026

ASN American Society of Nephrology | JASN Journal of the American Society of Nephrology | KIDNEY360 Accessing Our World From Every Angle | CJASN Clinical Journal of the American Society of Nephrology

ASN Journal Disclosure Form

As per ASN journal policy, I have disclosed any financial relationships or commitments I have held in the past 36 months as included below. I have listed my Current Employer below to indicate there is a relationship requiring disclosure. If no relationship exists, my Current Employer is not listed.

H. Goebel reports the following:
Employer: University Hospital Cologne

I understand that the information above will be published within the journal article, if accepted, and that failure to comply and/or to accurately and completely report the potential financial conflicts of interest could lead to the following: 1) Prior to publication, article rejection, or 2) Post-publication, sanctions ranging from, but not limited to, issuing a correction, reporting the inaccurate information to the authors' institution, banning authors from submitting work to ASN journals for varying lengths of time, and/or retraction of the published work.

Name: Heike Goebel
Manuscript ID: K360-2025-001311R1
Manuscript Title: he interplay between gut microbiota and diet-induced kidney protection
Date of Completion: March 2, 2026
Disclosure Updated Date: March 2, 2026

ASN
American Society of Nephrology
JASN
Journal of the American Society of Nephrology
KIDNEY360
Accessing Our World From Every Angle
CJASN
Clinical Journal of the American Society of Nephrology

ASN Journal Disclosure Form

As per ASN journal policy, I have disclosed any financial relationships or commitments I have held in the past 36 months as included below. I have listed my Current Employer below to indicate there is a relationship requiring disclosure. If no relationship exists, my Current Employer is not listed.

K. Hoyer-Allo reports the following:
Employer: University Hospital Cologne

I understand that the information above will be published within the journal article, if accepted, and that failure to comply and/or to accurately and completely report the potential financial conflicts of interest could lead to the following: 1) Prior to publication, article rejection, or 2) Post-publication, sanctions ranging from, but not limited to, issuing a correction, reporting the inaccurate information to the authors' institution, banning authors from submitting work to ASN journals for varying lengths of time, and/or retraction of the published work.

Name: Karla Johanna Ruth Hoyer-Allo
Manuscript ID: K360-2025-001311R3
Manuscript Title: The interplay between gut microbiota and diet-induced kidney protection
Date of Completion: May 4, 2026
Disclosure Updated Date: May 12, 2025

ASN Journal Disclosure Form

As per ASN journal policy, I have disclosed any financial relationships or commitments I have held in the past 36 months as included below. I have listed my Current Employer below to indicate there is a relationship requiring disclosure. If no relationship exists, my Current Employer is not listed.

L. Knieps reports the following:
Employer: University Hospital Cologne; and Other Interests or Relationships: Deutschlandstipendium at the University of Cologne, funded by Stiftung Maria Pesch.

I understand that the information above will be published within the journal article, if accepted, and that failure to comply and/or to accurately and completely report the potential financial conflicts of interest could lead to the following: 1) Prior to publication, article rejection, or 2) Post-publication, sanctions ranging from, but not limited to, issuing a correction, reporting the inaccurate information to the authors' institution, banning authors from submitting work to ASN journals for varying lengths of time, and/or retraction of the published work.

Name: Luisa Knieps
Manuscript ID: K360-2025-001311R1
Manuscript Title: The interplay between gut microbiota and diet-induced kidney protection
Date of Completion: March 6, 2026
Disclosure Updated Date: March 6, 2026

ASN American Society of Nephrology JASN Journal of the American Society of Nephrology KIDNEY360 Accessing Our World From Every Angle CJASN Clinical Journal of the American Society of Nephrology

ASN Journal Disclosure Form

As per ASN journal policy, I have disclosed any financial relationships or commitments I have held in the past 36 months as included below. I have listed my Current Employer below to indicate there is a relationship requiring disclosure. If no relationship exists, my Current Employer is not listed.

F. Koehler reports the following:
Employer: University Hospital of Cologne; Consultancy: Atriva Therapeutics GmbH; and Research Funding: Cologne Clinician Scientist Program (CCSP) / Faculty of Medicine / University of Cologne, funded by the Deutsche Forschungsgemeinschaft (DFG, German Research Foundation) (Project No. 413543196), Else Kröner-Fresenius-Stiftung, German Research Foundation under Germany's Excellence Strategy – EXC 2030: CECAD – Excellent in Aging Research - Project number 390661388, Koeln Fortune program / Faculty of Medicine, University of Cologne, Center for Molecular Medicine Cologne (CMMC).

I understand that the information above will be published within the journal article, if accepted, and that failure to comply and/or to accurately and completely report the potential financial conflicts of interest could lead to the following: 1) Prior to publication, article rejection, or 2) Post-publication, sanctions ranging from, but not limited to, issuing a correction, reporting the inaccurate information to the authors' institution, banning authors from submitting work to ASN journals for varying lengths of time, and/or retraction of the published work.

Name: Felix C. Koehler
Manuscript ID: K360-2025-001311R1
Manuscript Title: The interplay between gut microbiota and diet-induced kidney protection
Date of Completion: March 2, 2026
Disclosure Updated Date: March 2, 2026

ASN
American Society of Nephrology
JASN
Journal of the American Society of Nephrology
KIDNEY360
Accessing Our World From Every Angle
CJASN
Clinical Journal of the American Society of Nephrology

ASN Journal Disclosure Form

As per ASN journal policy, I have disclosed any financial relationships or commitments I have held in the past 36 months as included below. I have listed my Current Employer below to indicate there is a relationship requiring disclosure. If no relationship exists, my Current Employer is not listed.

F. Lopes has nothing to disclose.

I understand that the information above will be published within the journal article, if accepted, and that failure to comply and/or to accurately and completely report the potential financial conflicts of interest could lead to the following: 1) Prior to publication, article rejection, or 2) Post-publication, sanctions ranging from, but not limited to, issuing a correction, reporting the inaccurate information to the authors' institution, banning authors from submitting work to ASN journals for varying lengths of time, and/or retraction of the published work.

Name: Francisco José Calheiros Craveiro Lopes
Manuscript ID: K360-2025-001311R2
Manuscript Title: The interplay between gut microbiota and diet-induced kidney protection
Date of Completion: March 30, 2026
Disclosure Updated Date: March 30, 2026

ASN
American Society of Nephrology
JASN
Journal of the American Society of Nephrology
KIDNEY360
Accessing Our World From Every Angle
CJASN
Clinical Journal of the American Society of Nephrology

ASN Journal Disclosure Form

As per ASN journal policy, I have disclosed any financial relationships or commitments I have held in the past 36 months as included below. I have listed my Current Employer below to indicate there is a relationship requiring disclosure. If no relationship exists, my Current Employer is not listed.

D. Martinez Martinez reports the following:
Employer: University of Hertfordshire; Consultancy: none; Ownership Interest: none; Research Funding: none; Honoraria: none; Patents or Royalties: none; Advisory or Leadership Role: none; Speakers Bureau: none; and Other Interests or Relationships: none.

I understand that the information above will be published within the journal article, if accepted, and that failure to comply and/or to accurately and completely report the potential financial conflicts of interest could lead to the following: 1) Prior to publication, article rejection, or 2) Post-publication, sanctions ranging from, but not limited to, issuing a correction, reporting the inaccurate information to the authors' institution, banning authors from submitting work to ASN journals for varying lengths of time, and/or retraction of the published work.

Name: Daniel Martinez Martinez
Manuscript ID: K360-2025-001311R3
Manuscript Title: The interplay between gut microbiota and diet-induced kidney protection
Date of Completion: May 5, 2026
Disclosure Updated Date: May 5, 2026

ASN American Society of Nephrology · JASN Journal of the American Society of Nephrology · KIDNEY360 Accessing Our World From Every Angle · CJASN Clinical Journal of the American Society of Nephrology

ASN Journal Disclosure Form

As per ASN journal policy, I have disclosed any financial relationships or commitments I have held in the past 36 months as included below. I have listed my Current Employer below to indicate there is a relationship requiring disclosure. If no relationship exists, my Current Employer is not listed.

R. Mueller reports the following:
Employer: Dept. II of Internal Medicine, University Hospital Cologne, University of Cologne; Dept. of Nephrology, University Hospital Duesseldorf, University Duesseldorf; Consultancy: Alnylam, Vifor, GSK, AICURIS, Vertex; Research Funding: Alnylam Pharmaceuticals; Honoraria: see consultancy; Patents or Royalties: Detechgene; and Advisory or Leadership Role: Scientific Advisory Board Santa Barbara Nutrients.

I understand that the information above will be published within the journal article, if accepted, and that failure to comply and/or to accurately and completely report the potential financial conflicts of interest could lead to the following: 1) Prior to publication, article rejection, or 2) Post-publication, sanctions ranging from, but not limited to, issuing a correction, reporting the inaccurate information to the authors' institution, banning authors from submitting work to ASN journals for varying lengths of time, and/or retraction of the published work.

Name: Roman-Ulrich Mueller
Manuscript ID: K360-2025-001311R1
Manuscript Title: The interplay between gut microbiota and diet-induced kidney protection
Date of Completion: March 4, 2026
Disclosure Updated Date: January 7, 2026

ASN Journal Disclosure Form

As per ASN journal policy, I have disclosed any financial relationships or commitments I have held in the past 36 months as included below. I have listed my Current Employer below to indicate there is a relationship requiring disclosure. If no relationship exists, my Current Employer is not listed.

T. Rabelink has nothing to disclose.

I understand that the information above will be published within the journal article, if accepted, and that failure to comply and/or to accurately and completely report the potential financial conflicts of interest could lead to the following: 1) Prior to publication, article rejection, or 2) Post-publication, sanctions ranging from, but not limited to, issuing a correction, reporting the inaccurate information to the authors' institution, banning authors from submitting work to ASN journals for varying lengths of time, and/or retraction of the published work.

Name: Ton J. Rabelink
Manuscript ID: K360-2025-001311R3
Manuscript Title: The interplay between gut microbiota and diet-induced kidney protection
Date of Completion: April 30, 2026
Disclosure Updated Date: April 30, 2026

ASN American Society of Nephrology | JASN Journal of the American Society of Nephrology | KIDNEY360 Accessing Our World From Every Angle | CJASN Clinical Journal of the American Society of Nephrology

ASN Journal Disclosure Form

As per ASN journal policy, I have disclosed any financial relationships or commitments I have held in the past 36 months as included below. I have listed my Current Employer below to indicate there is a relationship requiring disclosure. If no relationship exists, my Current Employer is not listed.

B. Schermer reports the following:
Employer: University Hospital Cologne

I understand that the information above will be published within the journal article, if accepted, and that failure to comply and/or to accurately and completely report the potential financial conflicts of interest could lead to the following: 1) Prior to publication, article rejection, or 2) Post-publication, sanctions ranging from, but not limited to, issuing a correction, reporting the inaccurate information to the authors' institution, banning authors from submitting work to ASN journals for varying lengths of time, and/or retraction of the published work.

Name: Bernhard Schermer
Manuscript ID: K360-2025-001311R1
Manuscript Title: The interplay between gut microbiota and diet-induced kidney protection
Date of Completion: March 9, 2026
Disclosure Updated Date: March 9, 2026

ASN American Society of Nephrology | JASN Journal of the American Society of Nephrology | KIDNEY360 Accessing Our World From Every Angle | CJASN Clinical Journal of the American Society of Nephrology

ASN Journal Disclosure Form

As per ASN journal policy, I have disclosed any financial relationships or commitments I have held in the past 36 months as included below. I have listed my Current Employer below to indicate there is a relationship requiring disclosure. If no relationship exists, my Current Employer is not listed.

G. Schwarz reports the following:
Employer: University of Cologne; Consultancy: Consultation to Sentynl Therapeutics Inc. , Solana Beach, CA 92075, USA; and Ownership Interest: CEO of Colbourne Pharmaceutical GmbH.

I understand that the information above will be published within the journal article, if accepted, and that failure to comply and/or to accurately and completely report the potential financial conflicts of interest could lead to the following: 1) Prior to publication, article rejection, or 2) Post-publication, sanctions ranging from, but not limited to, issuing a correction, reporting the inaccurate information to the authors' institution, banning authors from submitting work to ASN journals for varying lengths of time, and/or retraction of the published work.

Name: Guenter Schwarz
Manuscript ID: K360-2025-001311R2
Manuscript Title: The interplay between gut microbiota and diet-induced kidney protection
Date of Completion: March 25, 2026
Disclosure Updated Date: March 25, 2026

ASN Journal Disclosure Form

As per ASN journal policy, I have disclosed any financial relationships or commitments I have held in the past 36 months as included below. I have listed my Current Employer below to indicate there is a relationship requiring disclosure. If no relationship exists, my Current Employer is not listed.

M. Späth reports the following:
Employer: University Hospital of Duesseldorf, Kuratorium für Hämodialyse e. V.; Consultancy: Serb GmbH, Fresenius medical care; Honoraria: Serb GmbH, Fresenius medical care, AstraZeneca; and Advisory or Leadership Role: advisory boards: Serb GmbH, Fresenius medical care,.

I understand that the information above will be published within the journal article, if accepted, and that failure to comply and/or to accurately and completely report the potential financial conflicts of interest could lead to the following: 1) Prior to publication, article rejection, or 2) Post-publication, sanctions ranging from, but not limited to, issuing a correction, reporting the inaccurate information to the authors' institution, banning authors from submitting work to ASN journals for varying lengths of time, and/or retraction of the published work.

Name: Martin R. Späth
Manuscript ID: K360-2025-001311R1
Manuscript Title: The interplay between gut microbiota and diet-induced kidney protection
Date of Completion: March 9, 2026
Disclosure Updated Date: March 9, 2026

ASN American Society of Nephrology
JASN Journal of the American Society of Nephrology
KIDNEY360 Accessing Our World From Every Angle
CJASN Clinical Journal of the American Society of Nephrology

ASN Journal Disclosure Form

As per ASN journal policy, I have disclosed any financial relationships or commitments I have held in the past 36 months as included below. I have listed my Current Employer below to indicate there is a relationship requiring disclosure. If no relationship exists, my Current Employer is not listed.

S. Strubl reports the following:
Employer: University Hospital Cologne

I understand that the information above will be published within the journal article, if accepted, and that failure to comply and/or to accurately and completely report the potential financial conflicts of interest could lead to the following: 1) Prior to publication, article rejection, or 2) Post-publication, sanctions ranging from, but not limited to, issuing a correction, reporting the inaccurate information to the authors' institution, banning authors from submitting work to ASN journals for varying lengths of time, and/or retraction of the published work.

Name: Sebastian Strubl
Manuscript ID: K360-2025-001311R1
Manuscript Title: The interplay between gut microbiota and diet-induced kidney protection
Date of Completion: March 5, 2026
Disclosure Updated Date: March 5, 2026

ASN American Society of Nephrology
JASN Journal of the American Society of Nephrology
KIDNEY360 Accessing Our World From Every Angle
CJASN Clinical Journal of the American Society of Nephrology

ASN Journal Disclosure Form

As per ASN journal policy, I have disclosed any financial relationships or commitments I have held in the past 36 months as included below. I have listed my Current Employer below to indicate there is a relationship requiring disclosure. If no relationship exists, my Current Employer is not listed.

B. van den Berg reports the following:
Employer: Leiden University Medical Center

I understand that the information above will be published within the journal article, if accepted, and that failure to comply and/or to accurately and completely report the potential financial conflicts of interest could lead to the following: 1) Prior to publication, article rejection, or 2) Post-publication, sanctions ranging from, but not limited to, issuing a correction, reporting the inaccurate information to the authors' institution, banning authors from submitting work to ASN journals for varying lengths of time, and/or retraction of the published work.

Name: Bernard van den Berg
Manuscript ID: K360-2025-001311R2
Manuscript Title: The interplay between gut microbiota and diet-induced kidney protection
Date of Completion: March 26, 2026
Disclosure Updated Date: March 26, 2026
