# Supplementary material for: The Interplay between Gut Microbiota and Diet-Induced Kidney Protection
Source: Kidney360. 2026 May 26;7(9):1930–42. doi: 10.34067/KID.0000001219 (PMC13641456; doi:10.34067/KID.0000001219)
Supplement: Supplementary file 2 [file kidney360-7-1930-s002.pdf]

Supplemental File

Item number	FMD Day 1 S5159-E736	FMD Day 2-3 S5159-E738
**Proximate Content**		
Broth mix (g)	5.95	5.31
EVOO (g)	12.75	0.0
Essential fatty acid (g)	0.21	0.0
Vegetable mix (g)	14.88	0.0
Glycerol (g)	0.0	0.75
Hydrogel	56.7	94.96
Fiber (g)	5.0	0.0
Mineral (AIN-93G-MX), Teklad, TD 94046, ENVIGO	3.5	0.0
Vitamin (AIN-93-VX), Teklad, TD 94047, ENVIGO	1.0	0.0
**Metabolizable energy [MJ/kg][1]**	7.67	1.48
kcal Protein (%)	5.0	0.5
kcal Fat (%)	28.0	0.5
kcal Carbohydrates (%)	64.0	99.0

Supplemental Table 1: Content and metabolizable energy of the fasting mimicking diet (FMD) components used on day 1 and day 2/3. There are two different dietary components designated as day 1 and day 2-3 diet during FMD regimens, respectively. This is followed by the administration of the standard chow (Table S2) that was fed *ad libitum* for 4 days. FMD was produced by ssniff Spezialdiäten GmbH, Soest, Germany as previously described (1, 2). Specified item numbers are depicted. [1] The Atwater formula corresponding to 3.275 kcal/kg was used to calculated the metabolizable energy of the respective diets. Abbreviations: EVOO: Extra Virgin Olive Oil; FMD: Fasting Mimicking Diet; kcal: kilocalories.

Item number	SR<br>S5159-E720	Standard chow<br>V1554-330
**Proximate content**		
Crude protein (%)	11.9	19.3
Crude fat (%)	4.5	3.3
Crude fiber (%)	4.9	4.4
Crude ash (%)	3.2	6.0
Starch (%)	51.2	36.0
Sugar (%)	19.9	5.0
Dextrin (%)	0.0	0.0
Lysine (%)	1.09	1.04
Methionine (%)	0.0	0.37
Cysteine (%)	0.0	0.37
Valine (%)	0.79	1.04
Isoleucine (%)	0.79	0.84
Leucine (%)	1.07	1.64
Glutamic acid (%)	3.50	3.68
Calcium (%)	0.52	1.0
Phosphorus (%)	0.34	0.7
Sodium (%)	0.15	0.23
Magnesium (%)	0.07	0.22
Potassium (%)	0.48	0.73
**Metabolizable energy [MJ/kg][1]**	16.0	16.4
kcal Protein (%)	12.0	23.0
kcal Fat (%)	11.0	10.0
kcal Carbohydrates (%)	77.0	67.0

Supplemental Table 2: Proximate content and metabolizable energy of a diet depleted in the sulfur-containing amino acids (SR) and the standard chow used for dietary preconditioning. All diets were obtained from ssniff Spezialdiäten GmbH, Soest, Germany with the respective item numbers given. [1] The Atwater formula corresponding to 3.275 kcal/kg was used to calculated the metabolizable energy of the respective diets. Abbreviations SR: a diet depleted in the sulfur-containing amino acids.

Weight loss	Compared to baseline prior renal ischemia-reperfusion injury
0 points	no weight loss
1 point	< 5%
5 points	5 - 10%
10 points	11-19%
20 points	20%

Appearance	
0 points	smooth fur, clean body-openings, clear and shiny eyes
1 point	shaggy fur, dull skin and/or scaly skin, hair loss without skin pathologies, small excoriations (superficial and locally circumscribed without rush or swelling)
10 points	clotty fur and/or clotty body-openings, dull eyes, head tilting, body tilting, moderate excoriations/skin pathologies (with rush or swelling), impaired wound healing
20 points	severe excoriations or skin pathologies (severe rush, swelling, purulent discharge, no wound-healing), abscess, tense abdomen, distended abdomen, paralysis)

Activity	
0 points	normal appearance (flight reflex, nest building, fur cleaning, movement in cage)
5 points	decelerated movement and/or occasional stereotypies
10 points	self-isolation, occasional lethargy with half-closed eyes, severe hyperactivity, severe stereotypies (e.g. spinning-around) and/or impairment of coordination (e.g. staggering and/or reeling)
20 points	self-aggression/ -mutilation, apathy (fully closed eyes)

Total sum	
< 10 points	continue experiment according to protocol
11-19 points	close observation and consultation with experimental supervisor
> 20 points	immediate sacrifice of the animal

Supplemental Table 3: Governmental approved recovery score after renal IRI based on weight loss, appearance and activity. Postoperative heath status after renal IRI was examined daily after renal IRI. The recovery score was compiled based on the “Belastungskataloge zur Bewertung von Tierversuchen” of the „Forum Tierversuche in der Forschung“ and was approved by the Landesamt für Verbraucherschutz und Ernährung NRW (LAVE) (AZ: 2019.A345). Abbreviation: IRI: Ischemia Reperfusion Injury.

Supplemental Figure Legend

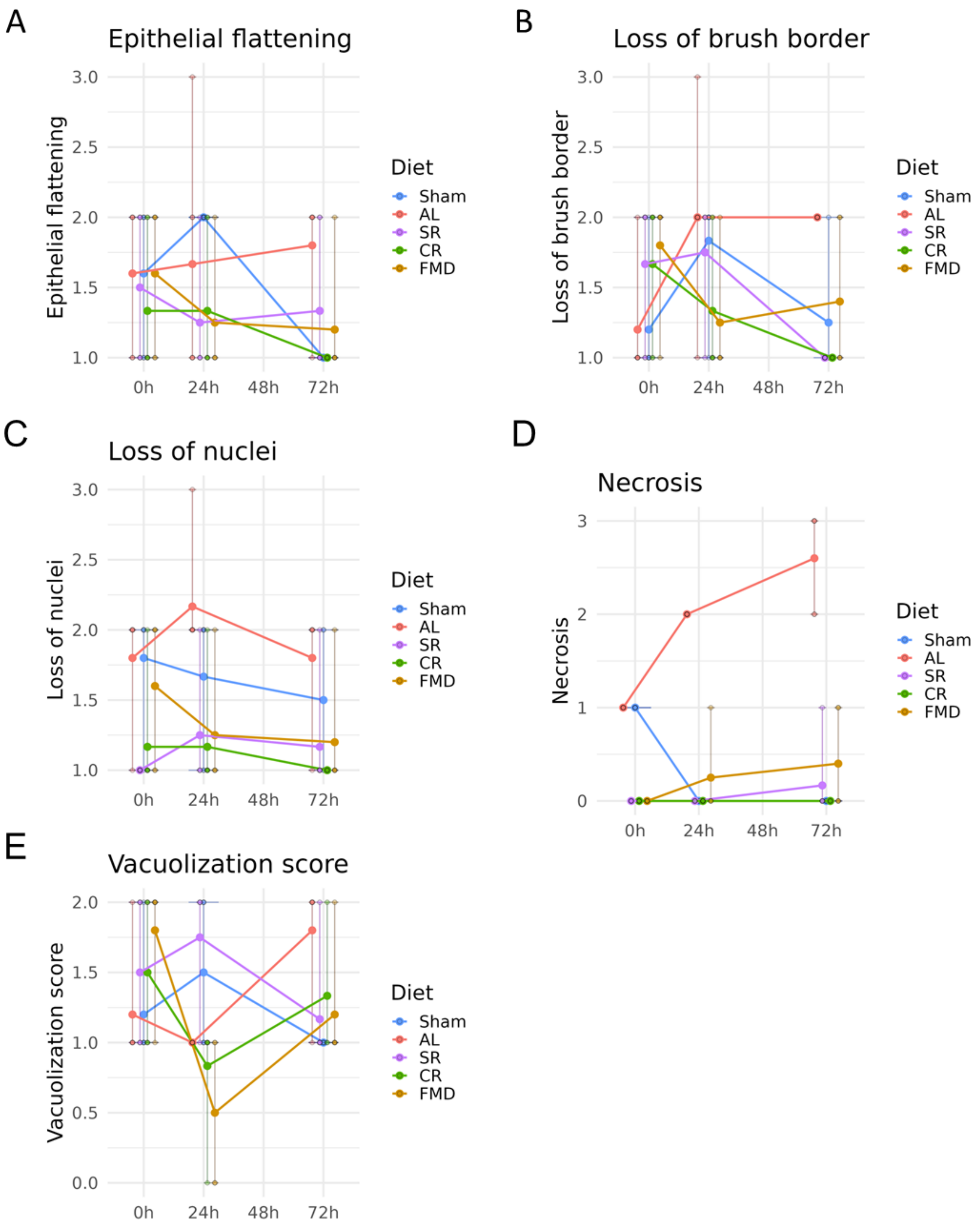

Supplemental Figure 1: Quantification of the items (A) epithelial flattening, (B) loss of brush borders, (C) loss of nuclei, (D) necrosis and (E) vacuolization of the used histologic damage score established in a blinded manner in our model of renal IRI. (A-E) Minimum and maximum are given as a transparent line with each animal being depicted

as a single dot. n = 4-6 mice per group per time point. Abbreviations: AL: *ad libitum*, CR: caloric restriction, SR: a diet depleted in the sulfur-containing amino acids, FMD: fasting mimicking diet, IRI: ischemia reperfusion injury.

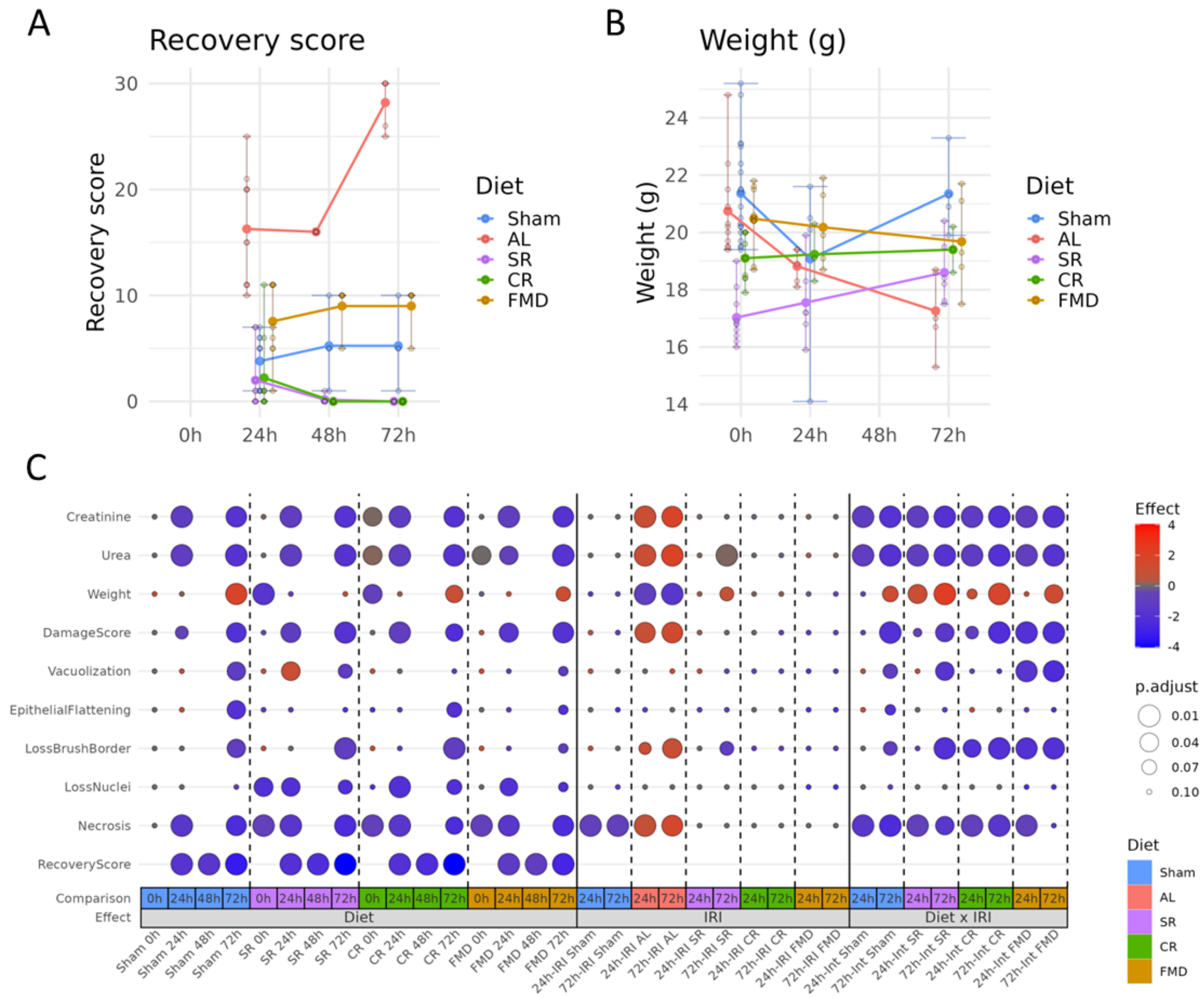

Supplemental Figure 2: A governmental recovery score after renal IRI using the three items weight loss, activity and appearance (Table S1) was used to quantify health following renal IRI. (A) Overall recovery score and its respective sub-item (B) weight are given. (A, B) Minimum and maximum are given as a transparent line with each animal being depicted as a single dot. n = 4-6 mice per group per time point. (C) Overview of statistical outcomes regarding kidney function, general health as reflected by a governmental approved recovery score and of the histopathological damage score together with its subitems given as diet effects, IRI effects in addition to their interaction (Diet x IRI). Continuous outcomes (creatinine, urea, weight, recovery-score), diet effects and IRI effects were evaluated using Welch two-sample t-tests; effect sizes are reported as mean differences between groups. Diet × IRI interaction effects were evaluated using a linear model including an interaction term (y ~

Diet * Time), with interaction effect sizes interpreted as difference-in-differences. Ordinal histology / score outcomes (histopathological damage score, its respective items vacuolization, epithelial flattening, loss of brush borders, loss of nuclei, necrosis) diet effects and IRI effects were evaluated using Wilcoxon rank-sum tests; effect sizes are reported as median differences between groups. Diet × IRI interaction effects were evaluated using a rank-based linear model (rank(y) ~ Diet * Time), with interaction effect sizes interpreted as difference-in-differences of medians. Abbreviations: AL: *ad libitum*, CR: caloric restriction, SR: a diet depleted in the sulfur-containing amino acids, FMD: fasting mimicking diet, IRI: ischemia reperfusion injury.

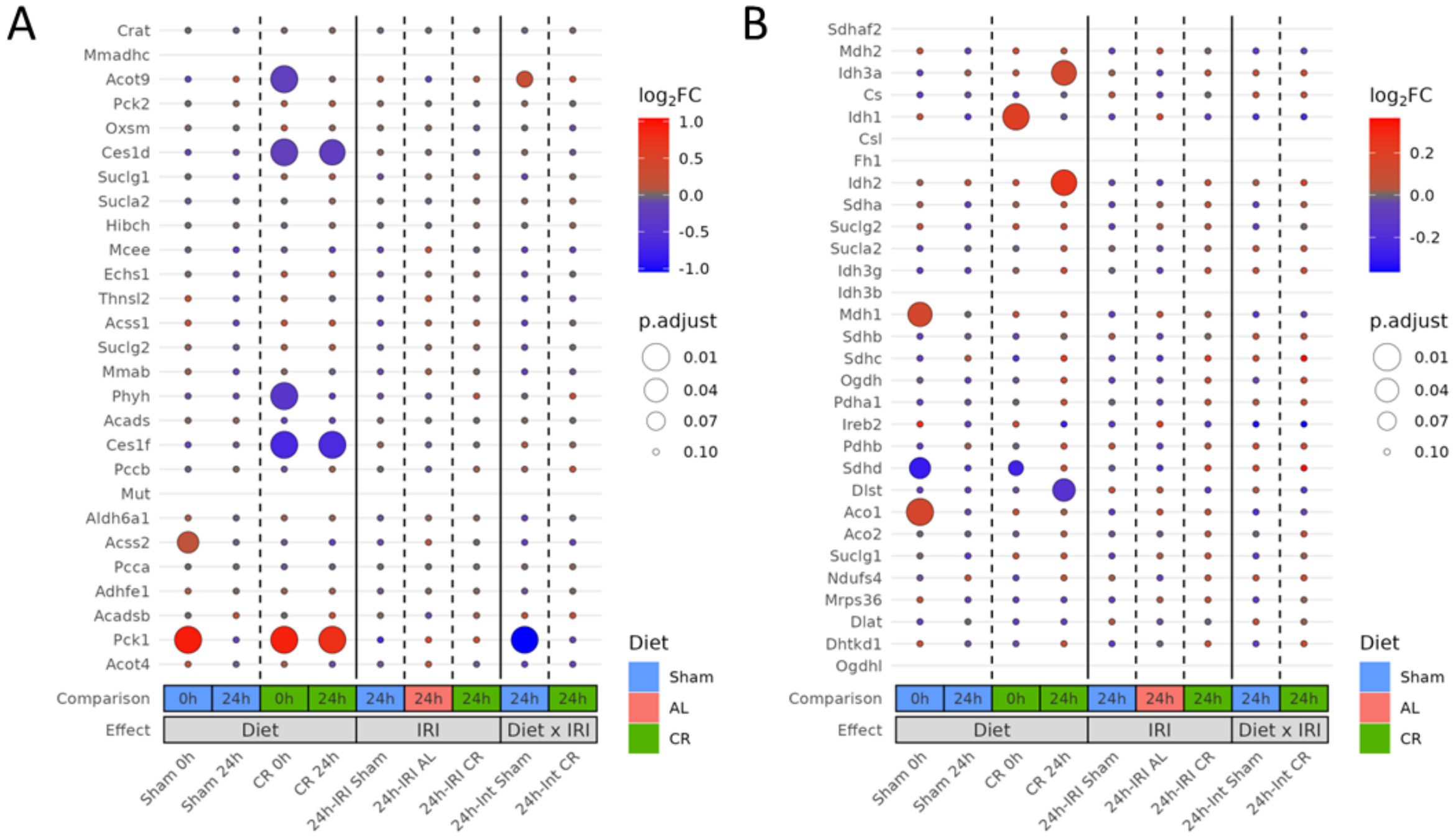

Supplemental Figure 3: Recapitulation of diet-induced metabolic reprogramming on the protein level in kidneys of protected mice. (A, B) Protein-level dot plots showing protein differential expression results as detected by bulk proteomics for (A) short-chain fatty acid (SCFA) metabolism-related genes and (B) tricarboxylic acid (TCA) cycle-related genes across the same set of contrasts (Diet at 0 h, Diet at 24 h, IRI effect at 24 h within diet, and the Diet × IRI interaction at 24 h). Dot color indicates log2 fold change for each gene in the indicated contrast; dot size indicates the BH-adjusted p value from the corresponding test. n = 4 mice per group per time point.

Supplemental References

1. Brandhorst S, Choi IY, Wei M, Cheng CW, Sedrakyan S, Navarrete G, et al. A Periodic Diet that Mimics Fasting Promotes Multi-System Regeneration, Enhanced Cognitive Performance, and Healthspan. *Cell Metab.* 2015;22(1): 86-99 doi:10.1016/j.cmet.2015.05.012.

2. Koehler FC, Fu CY, Späth MR, Hoyer-Allo KJR, Bohl K, Göbel H, et al. A systematic analysis of diet-induced nephroprotection reveals overlapping changes in cysteine catabolism. *Transl Res.* 2022;doi:10.1016/j.trsl.2022.02.003.
